# Regional Homogeneity Abnormalities and Its Correlation With Impulsivity in Male Abstinent Methamphetamine Dependent Individuals

**DOI:** 10.3389/fnmol.2021.810726

**Published:** 2022-01-20

**Authors:** Yanan Zhou, Qianjin Wang, Honghong Ren, Xuyi Wang, Yanhui Liao, Zhi Yang, Yuzhu Hao, Yunfei Wang, Manyun Li, Yuejiao Ma, Qiuxia Wu, Yingying Wang, Dong Yang, Jiang Xin, Winson Fu Zun Yang, Long Wang, Tieqiao Liu

**Affiliations:** ^1^National Clinical Research Center for Mental Disorders, and Department of Psychiatry, The Second Xiangya Hospital of Central South University, Changsha, China; ^2^Hunan Key Laboratory of Psychiatry and Mental Health, Changsha, China; ^3^Department of Psychiatry, Hunan Brain Hospital (Hunan Second People’s Hospital), Changsha, China; ^4^Department of Psychiatry, Sir Run Run Shaw Hospital, School of Medicine, Zhejiang University, Hangzhou, China; ^5^Laboratory of Psychological Heath and Imaging, Shanghai Mental Health Center, Shanghai Jiao Tong University School of Medicine, Institute of Psychological and Behavioral Science, Shanghai Jiao Tong University, Shanghai, China; ^6^School of Computer Science and Engineering, Central South University, Changsha, China; ^7^Department of Psychological Sciences, College of Arts & Sciences, Texas Tech University, Lubbock, TX, United States; ^8^Department of Psychiatry, Sanming City Taijiang Hospital, Sanming, China

**Keywords:** methamphetamine, impulsivity, regional homogeneity, resting state, functional magnetic resonance image

## Abstract

Methamphetamine (MA) use affects the brain structure and function. However, no studies have investigated the relationship between changes in regional homogeneity (ReHo) and impulsivity in MA dependent individuals (MADs). The aim of this study was to investigate the changes of brain activity under resting state in MADs and their relationship to impulsivity using ReHo method. Functional magnetic resonance imaging (fMRI) was performed to collect data from 46 MADs and 44 healthy controls (HCs) under resting state. ReHo method was used to investigate the differences in average ReHo values between the two groups. The ReHo values abnormalities of the brain regions found in inter-group comparisons were extracted and correlated with impulsivity. Compared to the HCs, MADs showed significant increased ReHo values in the bilateral striatum, while the ReHo values of the bilateral precentral gyrus and the bilateral postcentral gyrus decreased significantly. The ReHo values of the left precentral gyrus were negatively correlated with the BIS-attention, BIS-motor, and BIS-nonplanning subscale scores, while the ReHo values of the postcentral gyrus were only negatively correlated with the BIS-motor subscale scores in MADs. The abnormal spontaneous brain activity in the resting state of MADs revealed in this study may further improve our understanding of the neuro-matrix of MADs impulse control dysfunction and may help us to explore the neuropathological mechanism of MADs related dysfunction and rehabilitation.

## Introduction

Methamphetamine (MA), commonly known as “ice,” is an amphetamine-type stimulant (ATS) that is one of the most abused new drugs in the world (Shadloo et al., 2017). According to the 2019 China Drug Situation Report released in 2020, MA users in China accounted for 55.2 percent of its 2.148 million registered drug users, making it the largest drug user in the country by far, (China National Narcotic Control Commission, 2020). In addition to the high prevalence of MA use, the high recurrence rates of MA dependent individuals (MADs) exacerbate the problem, creating a huge public health burden worldwide (Jiang et al., 2021). Chronic use of MA has been associated with a variety of physical and mental health problems (e.g., cardiovascular disease, depression) (Schwarzbach et al., 2020; Jiang et al., 2021), daily dysfunction (e.g., impulsivity) (Moallem et al., 2018; Wang et al., 2020) and neurocognitive dysfunction (Basterfield et al., 2019; Mizoguchi and Yamada, 2019), contributing to a considerable global disease burden. Although increasing studies have been conducted on the treating MA use disorders, such as pharmacotherapy (Chan et al., 2019), psychotherapy (Harada et al., 2018), and repetitive transcranial magnetic stimulation (rTMS) (AshaRani et al., 2020), the effectiveness in reducing MA recurrence remains unsatisfactory.

A challenge for treating MA abuse is recurrence during abstinence (Li X. et al., 2019). Although many factors contribute to recurrence of MADs, a possible key predictor is impulsivity (Ahn et al., 2016; Vassileva and Conrod, 2019). Impulsivity is defined as a predisposition toward rapid, unplanned reactions to internal or external stimuli with diminished regard to their negative consequences to themselves or others (Ahn et al., 2016; Psederska et al., 2021). It is considered a key etiological factor in current conceptualizations of substance use disorder (SUD). Moreover, self-reported impulsivity is a strong predictor of poor treatment response (Fernie et al., 2010; Winhusen et al., 2013). Although MA dependence is associated with many neuropsychiatric and behavioral problems, impulsivity has been studied extensively because of its purported importance in initiation and escalation of drug use and the probability of recurrence (Schwartz et al., 2010).

One of the popular methods of investigating impulsivity in MA patients is through resting-state functional magnetic resonance imaging (rs-fMRI). Rs-fMRI is a powerful tool that measures brain activities by detecting changes in blood-oxygen-level-dependent (BOLD) signals in the resting brain (Park et al., 2019). It has been widely used recently because it does not require the involvement of any specific task in the scanner and could reveal the neural substrates of task-independent processes in diseases (Xie et al., 2021). A specific methodology in rs-fMRI is regional homogeneity (ReHo). ReHo evaluates signal synchronization by calculating the consistency of temporal variations of BOLD signals within local brain regions (Liu et al., 2019c), and has been used in many neuropsychiatric disorders (Li H. et al., 2019; Ma et al., 2019), but rarely in addiction (Liao et al., 2012; Qiu et al., 2013). Over the past few decades, several neuroimaging studies have assessed the relationship between abnormal brain function and abnormal behaviors (such as impulsivity) in MADs, but the results have been inconsistent. For instance, abstinent MADs showed less frontal activation during cognitive control compared to healthy controls (HCs) (Salo et al., 2013; Weafer et al., 2020), and showed less delay discounting activation than HCs in the bilateral precuneus, right caudate, anterior cingulate cortex (ACC), and dorsolateral prefrontal cortex (DLPFC) (Hoffman et al., 2008). In contrast, abstinence MADs have greater activation of MA-related cues in the ventral striatum and medial frontal cortex (Malcolm et al., 2016). Moreover, few functional magnetic resonance imaging (fMRI) studies have focused on the relationship between impulsivity and brain dysfunction.

In this study, we used the ReHo method to study the difference in spontaneous brain activity between MADs and HCs in the resting state. Based on our previous studies (Xie et al., 2021), we hypothesized that ReHo values in the resting state would differ in the relevant brain regions between MADs and HCs. We also hypothesized that differences in ReHo values might be related to impulsivity.

## Materials and Methods

### Participants

One hundred Han male participants (50 MADs and 50 HCs, aged 18 – 45, completion of at least 6 years of formal education; fluency in Chinese; right-handed) were enrolled in this study. Data for 3 MADs (2 had contraindications to MRI and 1 had abnormal scan) and 6 HCs (3 were lost to follow-up, 1 had contraindication to MRI, and 2 had abnormal scans) were excluded. Meanwhile, one MADs with maximal head motion exceeding 2 mm or rotations over 2° was excluded from further analysis. A total of 46 MADs and 44 HCs were included in the final analysis. MADs were recruited from the Kangda Voluntary Drug Rehabilitation Centers in Changsha, Hunan Province, while drug-free HCs were recruited *via* local community advertisements. All the MADs were diagnosed with MA use disorders per DSM-5 by two trained senior psychiatrists using the Structured Clinical Interview (SCID) (First et al., 2002). All the MADs were required not to use any psychoactive substances other than tobacco, including alcohol, at least 48 h before the MRI scan. In order to reduce the effects of different stages of abstinence on brain cognition, MADs were assessed 3 months after abstinence, during which time MADs used drugs. Participants were excluded if they met any of following criteria: (1) had any general medical condition or neurological disorders that could confound brain function; (2) had a history of severe head injury with skull fracture or loss of consciousness of more than 10 min; (3) had any current or previous psychiatric disorder or family history of psychiatric disorder; and (4) had contraindications for MRI (including implanted metallic devices or ferromagnetic material or claustrophobia).

### Clinical Assessment

All the participants completed the following self-report scales; all the instruments have good reliability and validity.

#### General Information

Basic demographic information included age, height, weight, education, marital status, employment, and income. We also reported the drinking, smoking, and betel use status in the two groups.

#### Impulsivity

The level of impulsivity was measured using the Barratt Impulsivity Scale 11 Edition (BIS-11), which is the most extensive self-report scale for this purpose (Subramanian et al., 2020). The Chinese version of BIS-11 was used to measure the cognitive impulsiveness, motor impulsiveness and non-planning impulsiveness of MADs. Items 4, 5, 13, 14, 15, 16, 17, 19, 20, 21, and 26 were reverse scored (Yao et al., 2007). The whole scale consists of 30 items, using a 5-point Likert scale for each item with a higher total score indicating stronger impulsivity (Patton et al., 1995).

### Magnetic Resonance Imaging Data Acquisition

Magnetic resonance imaging data of all participants were acquired in the resting condition using a 3.0T MRI scanner (Siemens Skyra, Munich) equipped with a 16-channel head coil at the Magnetic Resonance Imaging Center of Hunan Children’s Hospital, Changsha, China. None of the participants were taking any medications on the day of the MRI scan. Participants were instructed to remain awake and still in supine position with eyes closed. During the scanning, foam pads and earplugs were used to restrain head motion and to attenuate noise. Anatomical T1-weighted MRI data were acquired using a 3D magnetization preparing rapid acquisition gradient echo sequence with the following parameters: repetition time (TR) = 2,530 ms, echo time (TE) = 2.98 ms, flip angle = 7°, field of view = 256 × 256 mm, slice thickness = 1 mm, slice gap = 0 mm, voxel size = 1 × 1 × 1 mm^3^, number of slices = 176, and scanning time = 363 s. Functional images were obtained using a gradient echo-planar imaging (EPI) sequence with the following parameters: TR = 2,000 ms, TE = 30 ms, flip angle = 78°, field of view = 224 × 224 mm, slice thickness = 3.5 mm, slice gap = 0.7 mm, voxel size = 3.5 × 3.5 × 3.5 mm^3^, number of slice = 33, and scanning time = 488 s.

### Data Preprocessing

Functional MRI data were preprocessed according to standard procedures with the Data Processing Assistant for Resting-State fMRI (DPARSF, version 4.1) (Chao-Gan and Yu-Feng, 2010),^1^ running in MATLAB (version R2013b, The MathWorks, Inc., Natick, MA, United States) (Yan et al., 2016). Functional MRI data preprocessing consisted of the following steps: (1) remove the first 10 time points in case of unstable signal quality, (2) perform slice-timing adjustment, (3) perform realignment, excluding subjects with maximal head motion exceeding 2 mm or rotations over 2°, (4) remove the mean framewise displacement (FD) > 0.2 mm, (5) conduct spatial normalization to the EPI template of Montreal Neurological Institute (MNI) space by resampling to 3 mm × 3 mm × 3 mm, (6) remove linear detrending, (7) temporal band-pass filtering (0.01 – 0.1 Hz), (8) smooth at 8 mm full width at half maximum (FWHM), and (9) nuisance signals were regressed out, including Friston 24 head motion parameters, global signal, white matter signal, and cerebrospinal fluid signal.

### Regional Homogeneity Calculation

ReHo calculation was performed with the REST^2^ software. In short, this was achieved by calculating Kendall’s coefficient of concordance (KCC) of time series of a given voxel with those of its nearest 26 neighbors on a voxel-by-voxel basis (Zang et al., 2004). The KCC value was calculated to this voxel, and a separate KCC map was obtained for each participant. For standardization purpose, the individual ReHo maps were divided by their own global mean KCC within the whole-brain mask. The individual ReHo maps were then spatially smoothed with an 8 mm FWHM Gaussian kernel to reduce noise and residual differences in gyral anatomy.

### Statistical Analysis

All statistical analyses were performed using R version 3.5.3. Before statistical analysis, normality, and variance homogeneity were tested. Demographic and clinical data are compared between groups using the Chi-square test, Mann–Whitney U test, or Student’s *t*-test, When appropriate. Several one-way analyses of covariances (ANCOVAs) were used to analyze group differences in ReHo values among the pre-defined regions of interest (ROIs). Covariances used were smoking, drinking, and betel use. Bonferroni-correction was used at this stage to reduce the number of type-I errors. ANOVAs were also used to analyze group differences in impulsivity. Significant ReHo results were subsequently used as individual predictors in linear regression for impulsivity. Scores were mean centered before running linear regressions as interaction terms needed to be computed.

## Results

### Demographics and Clinical Characteristics

MADs were significantly older than HCs with lower weight and higher education levels (Table 1). More importantly, MADs smoked more (95.70%) than HCs (45.50%, *p* < 0.001), and had longer smoking duration (12.80 ± 4.85 years) than HCs (7.42 ± 6.73, *p* = 0.001). Although there were no significant differences in drinking (*p* = 0.052), MADs drank for a longer time (3.89 ± 5.44 years) compared to HCs (1.27 ± 3.01, *p* = 0.006). Finally, MADs also use more betel (67.40%) compared to HCs (40.90%, *p* = 0.021) and used betel for a longer duration (9.02 ± 5.17 years) as compared to HCs (3.78 ± 2.16, *p* < 0.001).

**TABLE 1:** Demographics and clinical characteristics of participants.

Variables *M* (SD) or *n* (%)		HC (*n* = 44)	MAD (*n* = 46)	*p*-value
Age		26.05 (6.81)	31.37 (5.51)	<0.001
Height		171.66 (5.41)	170.76 (6.65)	0.485
Weight		68.48 (9.40)	73.52 (11.74)	0.027
Education years		14.00 (3.20)	11.46 (3.15)	<0.001
Marital status (%)	Divorced	0 (0.00)	6 (13.00)	<0.001
	Married	10 (22.70)	27 (58.70)	
	Single	34 (77.30)	13 (28.30)	
Employment (%)	Employed	19 (43.20)	25 (54.30)	<0.001
	Freelance	4 (9.10)	12 (26.10)	
	student	21 (47.70)	0 (0.00)	
	Unemployed	0 (0.00)	9 (19.60)	
Income in Yuan (%)	<2,000	18 (40.90)	2 (4.30)	<0.001
	>10,000	2 (4.50)	8 (17.40)	
	2,000–5,000	10 (22.70)	18 (39.10)	
	5,000–10,000	14 (31.80)	18 (39.10)	
Smoking (%)	No	24 (54.50)	2 (4.30)	<0.001
	Yes	20 (45.50)	44 (95.70)	
Smoke years		7.42 (6.73)	12.80 (4.85)	0.001
Drinking (%)	No	30 (68.20)	21 (45.70)	0.052
	Yes	14 (31.80)	25 (54.30)	
Drink years		1.27 (3.01)	3.89 (5.44)	0.006
Betel use (%)	No	26 (59.10)	15 (32.60)	0.021
	Yes	18 (40.90)	31 (67.40)	
Betel years		3.78 (2.16)	9.02 (5.17)	<0.001

*M, mean; SD, standard deviation; n, number; %, percentage; MAD, methamphetamine dependent individual; and HC, health control.*

### Behavioral Results

There were no significant differences between MADs and HCs on BIS-attention at *F*_(1,85)_ = 0.11, *p* = 0.74, BIS-nonplanning at *F*_(1,85)_ = 0.29, *p* = 0.59, or BIS-motor at *F*_(1,85)_ = 1.58, *p* = 0.21.

### Neuroimaging Results

#### Regional Homogeneity

**FIGURE 1:**
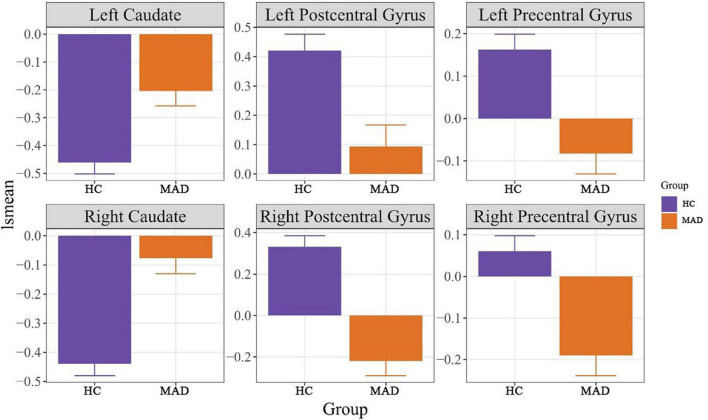
It displays the least square means (lsmeans) among HC and MAD for the significant regions of interest. HC, healthy control, MAD, methamphetamine dependent individual.

There were significant differences between MADs and HCs on ReHo values of the left caudate at *F*_(1,85)_ = 14.79, *p* = 0.017, right caudate at *F*_(1,85)_ = 30.00, *p* < 0.001, left postcentral gyrus at *F*_(1,85)_ = 31.96, *p* < 0.001, left precentral gyrus at *F*_(1,85)_ = 39.21, *p* = 0.0087, right postcentral gyrus at *F*_(1,85)_ = 16.73, *p* < 0.001, and right precentral gyrus at *F*_(1,85)_ = 17.06, *p* = 0.0089 after Bonferroni correction. See Figure 1 for more details.

#### Regression Analysis

**FIGURE 2:**
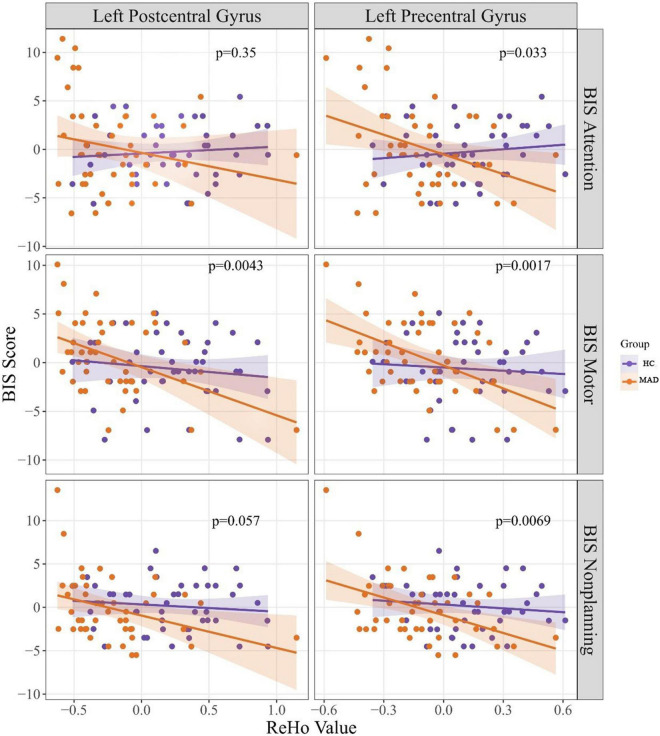
It presents the correlation between BIS scores and ReHo values. The *p*-values presented in this figure were *p*-values of the regression model. BIS, Barratt Impulsivity Scale, ReHo, regional homogeneity, HC, healthy control, and MAD, methamphetamine dependent individual.

Regression analysis of predicting BIS from ReHo values revealed a significant model for left postcentral gyrus for predicting BIS-motor at *F*_(3,86)_ = 4.72, *p* = 0.0043. Although there was a Group × ReHo interaction where higher left postcentral gyrus ReHo values in the MAD group was associated with lower BIS-motor scores, it only approached significance (*B* = −3.74, *p* = 0.074). There was also a significant model for ReHo values of the left precentral gyrus at *F*_(3,86)_ = 4.32, *p* = 0.0069. Although there was a Group × ReHo interaction where higher left precentral gyrus ReHo values in the MAD group was associated with lower BIS-nonplanning scores, it only approached significance (*B* = −5.35, *p* = 0.061). ReHo values of the left precentral gyrus predicted BIS-motor scores at *F*_(3,86)_ = 4.32, *p* = 0.001. There was a Group × ReHo interaction where higher left precentral gyrus ReHo values in the MAD group was associated with lower BIS-motor scores (*B* = −6.75, *p* = 0.034). ReHo values of the left precentral gyrus also predicted BIS-attention scores at *F*_(3,86)_ = 3.03, *p* = 0.033. There was a Group × ReHo interaction where higher left precentral gyrus ReHo values in the MAD group was associated with lower BIS-attention scores (*B* = −8.29, *p* = 0.017). There were no other significant models of ReHo values predicting BIS scores. See Figure 2 for more details.

## Discussion

To our knowledge, this is the first study to explore the relationship between ReHo values of local spontaneous brain activity and impulsivity in MADs. Compared with HCs, MADs showed significantly increased ReHo values in bilateral caudate, and decreased ReHo values in the bilateral postcentral gyrus, and the bilateral precentral gyrus. We further found that the ReHo values of the left precentral gyrus were negatively correlated with the BIS-attention, BIS-motor, and BIS-nonplanning subscale scores, while the ReHo values of the left postcentral gyrus were only negatively correlated with the BIS-motor subscale scores in MADs.

Compared to one of our previous studies, this study focused on the relationship between the abnormal ReHo values of the brain ROIs in MADs and the impulsivity of MADs (Xie et al., 2021). Moreover, most previous studies have focused on exploring the relationship between low-frequency fluctuation (ALFF) (Wang et al., 2013; Liu Y. et al., 2020), fractional amplitude of low frequency fluctuation (fALFF) (Chu et al., 2014; Wang et al., 2017) or structural MRI (Huang et al., 2020; Meade et al., 2020) and clinical variables, rather than ReHo. Furthermore, some studies on ReHo have focused on other addicts (Huang et al., 2020; Meade et al., 2020) and psychiatric patients (Liu P. et al., 2020; Shan et al., 2021), but not on MADs.

The striatum is a continuous mass structure composed of the caudate and putamen (Lerner et al., 2012), which can directly participate in rewards, movement control, regulation, and decision-making, especially action selection and initiation (de la Fuente-Fernández et al., 2002; Liu et al., 2019a). In this study, the increase in the bilateral striatum ReHo of MADs indicates the importance of their spontaneous neural activity in resting brain of MADs. This is consistent with our previous findings that ReHo was increased in the bilateral striatum of MADs compared to HCs (Xie et al., 2021; Yang et al., 2021). To date, only a few rs-fMRI studies have used ReHo to explore spontaneous brain activity changes and synchronization in individuals with SUD. A comparative study in recurring heroin addicts found that ReHo in the right caudate of recurred heroin addicts increased (Chang et al., 2016). It should be noted that compared with HCs, the ReHo of the left dorsal striatum was reduced in codeine-containing cough syrups (CCS) dependent individuals, and was negatively correlated with the BIS-11 total scores and attentional impulsivity score (Qiu et al., 2013). The reason why our results were inconsistent with this study may be mainly due to the heterogeneity of clinical samples (i.e., different addictive substances, different data collection status, and different sample sizes). Thus, the different mechanisms underlying MA and codeine use may result in representation in the brain. In addition, the ReHo values may change during withdrawal. Using fALFF, it was found that the activity of spontaneous neurons in the caudate of smokers was enhanced (Feng et al., 2016). Meanwhile, structural MRI studies showed a significant increase in gray matter volume in the putamen and caudate nuclei of cocaine users compared to HCs (Ersche et al., 2011). Although the methodologies were different, these findings provide strong support for the theory that the bilateral striatum is a key region in addiction disorders, and our findings support this view.

It is worth noting that the ReHo values of the bilateral postcentral gyrus and the bilateral precentral gyrus were decreased in MADs. It is well known that precentral gyrus, as the primary motor cortex, is involved in somatosensory activity, while postcentral gyrus, as the primary sensory cortex, is involved in the initiation and regulation of spontaneous movement (Javed et al., 2021). However, their relevance to addiction should not be underestimated. Studies have shown that the sensory and motor cortex is involved in various stages of drug addiction (Yalachkov et al., 2010). Meanwhile, the activation of sensory and motor cortex induced by drug-related cues can predict relapse (Kosten et al., 2006). Previous studies have also shown that the ReHo values of MA-associated psychosis (MAP) decreases in the left postcentral gyrus (Yang et al., 2021). Moreover, studies of nicotine addicts have shown a significant increase in the ReHo values of paracentral lobule after 2 weeks of abstinence (Mo et al., 2018). More interestingly, the study further found that the ReHo values of the left precentral gyrus were negatively correlated with the BIS-attention, BIS-motor, and BIS-nonplanning subscales scores, while the ReHo values of the left postcentral gyrus were only negatively correlated with the BIS-motor subscale scores in MADs. It is not difficult to know from the results that the negative correlation with impulsivity is mainly concentrated in the left precentral gyrus, rather than the left postcentral gyrus, which is consistent with the previous research results. For example, a meta-analysis of cue-reactivity in behavioral addictions found increased neural activation in the precentral gyrus (Starcke et al., 2018). Similar results also exist in individual with SUD (such as cannabis, alcohol) (Cheng et al., 2014; Fede et al., 2020). The precentral gyrus is one of the four most important gyrus in the prefrontal cortex. The prefrontal cortex is known to be the higher-order association center of the brain as it is responsible for decision making, regulating impulsivity-related disorders such as drug addiction, reasoning, personality expression, maintaining social appropriateness, and other complex cognitive behaviors (Liu et al., 2019b; El-Baba and Schury, 2021). This may be one of the reasons why the precentral gyrus is more associated with impulsivity than the postcentral gyrus.

We observed that the ReHo values of the left precentral gyrus were negatively correlated with BIS-attention, BIS-motor, and BIS-nonplanning subscales scores in MADs. This may indicate that reduced ReHo values in the left precentral gyrus are more closely associated with impulsivity in MADs than in HCs. Also, as presented in this study, ReHo values of the left postcentral gyrus only negatively correlated with BIS-motor subscale scores in MADs. Different brain regions are thought to be preferentially involved in complementary aspects of cognitive control to produce appropriate behavior (Crunelle et al., 2014). The left precentral gyrus may be particularly involved in cognitive control of MAD behavior, behavioral aspect of impulsivity, and temporal impulsivity, while the left postcentral gyrus may only be particularly involved in the behavioral aspect of impulsivity. In addition, no correlation was observed between left precentral gyrus of HCs and BIS subscales scores in the current study. This may indicate that changes in the ReHo values of the left precentral gyrus cannot be a predictor of impulsivity in HCs. Therefore, the reduced ReHo values in the left precentral gyrus might indicate dysfunction in this region in MADs.

While our study provided new insights on the role of ReHo in the left precentral gyrus on impulsive behaviors, some limitations of our study need to be addressed. Firstly, the study was cross-sectional, and causal relationships between variables could not be rigorously evaluated. Therefore, longitudinal studies are needed to help address these issues in the future. Secondly, the study recruited only male MADs, and the results are not representative of brain changes in women. As previous studies have shown that gender is an influential factor of brain dysfunction in MADs, further study on female MADs is needed. Finally, as with all ReHo analyses, it is still debated what higher or lower ReHo represents in terms of biologic functions, or their interpretability toward behavioral change. As ReHo only measures local connectivity, and the brain consists of connected networks, other analyses are needed to supplement ReHo results. Furthermore, this study did not evaluate other factors related to the ReHo of MADs, such as chronic stress.

## Conclusion

In conclusion, this study found abnormal ReHo values of MADs in the bilateral striatum, the bilateral precentral gyrus, and the bilateral postcentral gyrus. These brain changes might have to do with self-control and external perception. We also observed that decreased ReHo values in the left precentral gyrus and the left postcentral gyrus were associated with impulsivity. These findings may help to illustrate the pathophysiological mechanism of MADs, particularly in impulsivity, and provide a basis for the urgent formulation of good clinical treatment and prevention strategies to prevent recurrence.

## Data Availability Statement

The data analyzed in this study is subject to the following licenses/restrictions: All data in the current study was stored in the PI’s affiliation, and is available from the corresponding authors on reasonable request and with completion of data user agreement. Requests to access these datasets should be directed to TL, liutieqiao123@csu.edu.cn.

## Ethics Statement

The studies involving human participants were reviewed and approved by the Ethics Committee of the Second Xiangya Hospital, Central South University (No. S095, 2013). The patients/participants provided their written informed consent to participate in this study.

## Author Contributions

TL and YL designed and supervised the study. YZ, YH, ML, YfW, and YyW collected the data. YZ, WY, QjW, and JX analyzed and interpreted the data. YZ and QjW drafted the manuscript. HR, XW, WY, YM, QxW, DY, LW, and TL revised the manuscript. All co-authors revised and approved the final version to be published.

## Conflict of Interest

The authors declare that the research was conducted in the absence of any commercial or financial relationships that could be construed as a potential conflict of interest.

## Publisher’s Note

All claims expressed in this article are solely those of the authors and do not necessarily represent those of their affiliated organizations, or those of the publisher, the editors and the reviewers. Any product that may be evaluated in this article, or claim that may be made by its manufacturer, is not guaranteed or endorsed by the publisher.

## Footnotes

1
http://www.restfmri.net

2
http://www.resting-fmri.sourceforge.net
